# Laparoscopic reversal of Hartmann procedure

**DOI:** 10.4103/0972-9941.28182

**Published:** 2006-12

**Authors:** Vishwanath Golash

**Affiliations:** Department of Surgery, Sultan Qaboos Hospital, Salalah, Sultanate of Oman

**Keywords:** Hartmann procedure, laparoscopy

## Abstract

**Background:**

The Hartmann procedure is a standard life-saving operation for acute left colonic complications. It is usually performed as a temporary procedure with the intent to reverse it later on. This reversal is associated with considerable morbidity and mortality by open method. The laparoscopic reestablishment of intestinal continuity after Hartmann procedure has shown better results in terms of decrease in morbidity and mortality.

**Materials and Methods:**

The laparoscopic technique was used consecutively in 12 patients for the reversal of Hartmann procedure in the last 3 years. The adhesiolysis and mobilization of the colon was done under laparoscopic guidance. The colostomy was mobilized and returned to abdominal cavity after tying the anvil in the proximal end. An end-to-end intracorporeal anastomosis was performed between the proximal colon and the rectum using the circular stapler.

**Results:**

Mean age of the patients was 40 years and the mean time of restoration of intestinal continuity was 130 days. Two patients were converted to open. The mean time of operation was 90 min. There were no postoperative complications and mortality. The mean hospital stay was 5 days.

**Conclusion:**

Laparoscopic reversal of Hartmann is technically safe and feasible.

## INTRODUCTION

The indications for the Hartmann procedure are greatly reduced in recent years due to changing trend in the management of acute diverticular disease by primary anastomosis, the main indications being intra-abdominal sepsis, trauma, volvulus and malignancy. Although done as a temporary procedure, the reversal rate is not very high; nearly 40% of patients will not have the reversal.[1,2] Reversal of Hartmann procedure is a major undertaking and due to its associated morbidity and mortality, many patients are left with permanent colostomy and many others elect not to have the reversal. The advances in laparoscopy and stapler anastomosis have made the reversal simpler and easier.

## MATERIALS AND METHODS

Between July 2002 and October 2005, the reversal of Hartmann procedure was performed in 15 patients. With our increasing experience in advanced laparoscopic surgery, the laparoscopic reversal of Hartmann procedure was attempted in 12 consecutive patients. Among the laparoscopy group, there were 8 male and 4 female patients in the age group of 18-65 years (mean age 40). Ninety-one percent of the Omani population is below the age of 50 years and population over 60 years is 4.9%. Due to the vast majority of population being young, the number of patients seen with cancer of colon and diverticular disease is low, but the incidence of road traffic accidents is high. In 12 patients for laparoscopic closure, the Hartmann procedure was performed for perforated diverticular disease with intra-abdominal sepsis in 6 patients, traumatic rupture of left colon and rectum following road traffic accident in 4 patients, stricture left colon at the site of previous anastomosis in 1 and obstructed cancer of left colon in another patient. Eleven patients had their Hartmann procedure performed by conventional laparotomy and 1 patient by laparoscopy. The diseased bowel was excised at the time of initial surgery. Few long nonabsorbable monofilament sutures were left on the rectal stump for identification later at the time of reversal. The time of reversal varied from 70 to 220 days (mean of 130 days). A routine barium enema was done in all patients with diverticular disease and cancer colon through the colostomy and also through the rectal stump before the reversal to rule out the residual pathology in the proximal colon and to assess the rectal segment. Barium studies showed diverticulae in the segment of distal segment of colon, apparently incomplete excision at previous operation. Colonoscopy/flexible sigmoidoscopy are alternatives to barium studies but until further prospective data are available, no firm recommendation can be made. Barium studies and endoscopy are complementary to each other. The proximal bowel and the rectal stump were prepared by mechanical cleansing prior to surgery. All patients received single dose of ceftrioxone and metronidazole at the time of induction of anesthesia. Further mobilization of the colon was done laparoscopically and through the colostomy wound. The adhesions varied from mild to severe requiring adhesiolysis. The localization and mobilization of rectal stump was straight forward in 5 patients, difficult in 5 patients. In 2 patients - one with obstructed left colon cancer and the other, a morbid obese lady with previous perforated diverticular disease - the adhesions were so severe that it was not possible to access the rectal stump and were converted to open. The operation time varied from 65-180 min (mean 90).

### Technique

**Figure 1:**
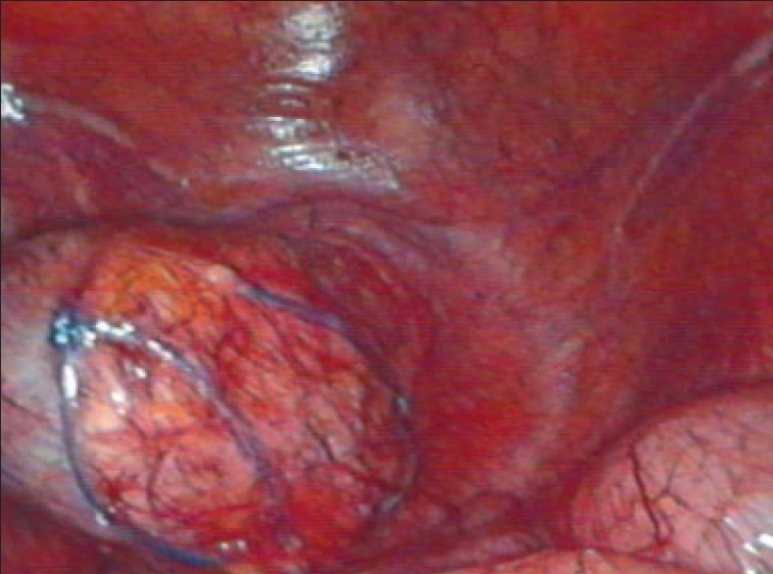
Recatal stump with bougie

**Figure 2:**
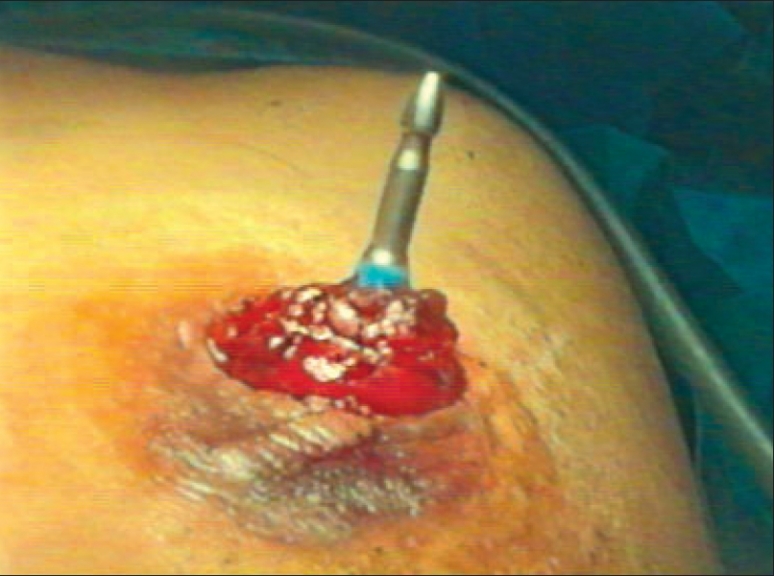
Anvil inserted and tied in the colostomy

**Figure 3:**
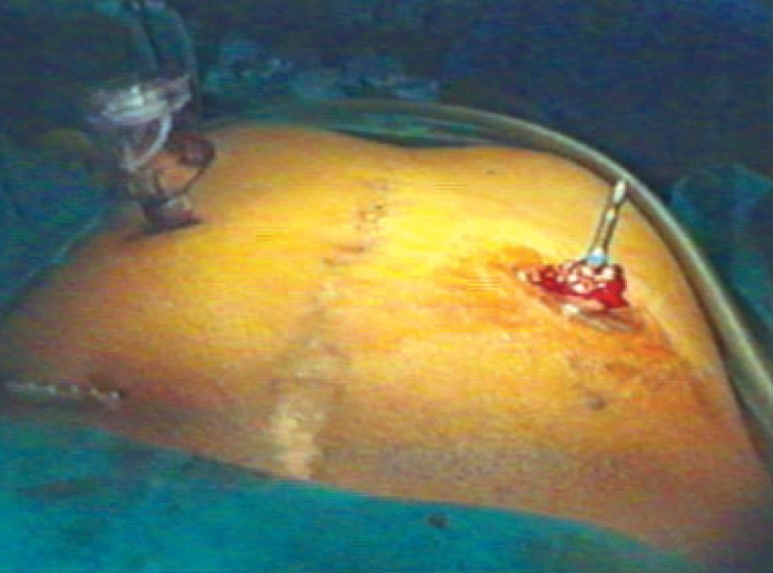
Port positions

**Figure 4:**
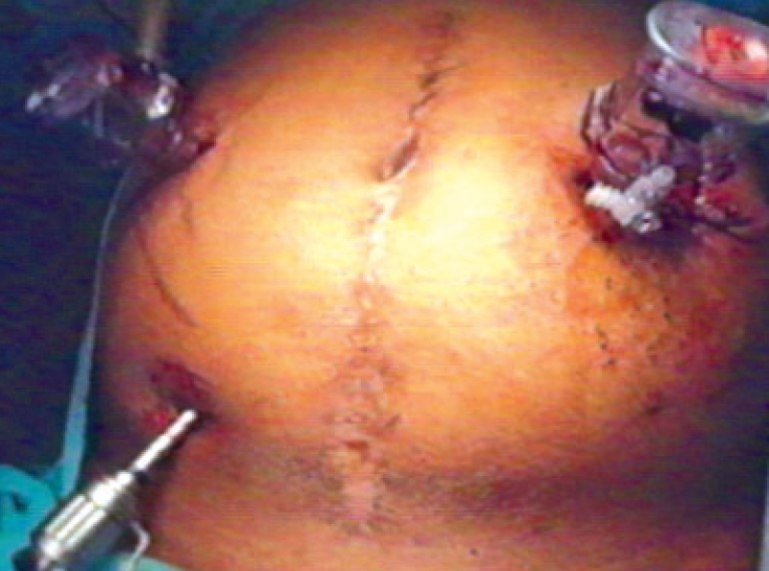
Utilizing the colostomy site for port

**Figure 5:**
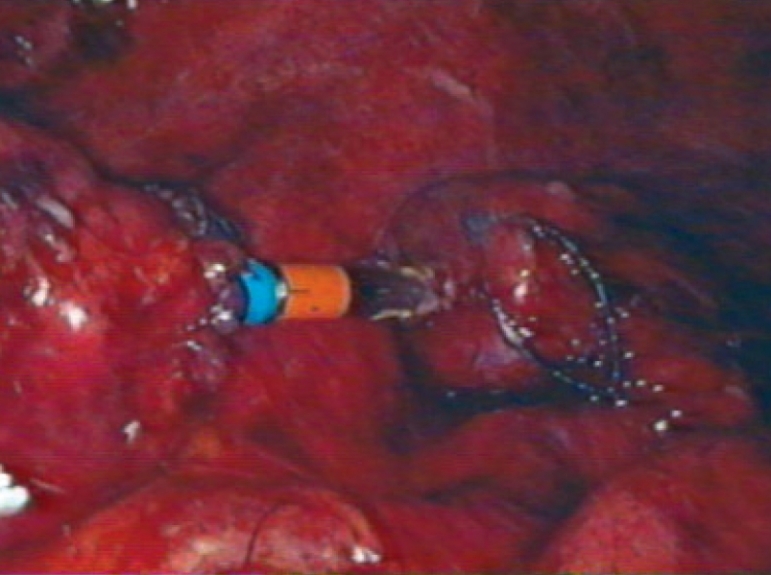
Intracorporeal stapler anstomosis

A diagnostic laparoscopy was done first to assess the feasibility of the procedure. A 10 mm port in right upper quadrant (optical port) and 5 mm port in the right iliac fossa were inserted in the midclavicular line. The adhesiolysis was performed and rectal stump was identified. The sigmoidoscope was inserted per rectally as a bougie to help in localizing the rectal stump [Figure 1]. Generally not much dissection and mobilization was done for the rectal stump; only adhesions were cleared anteriorly for the anastomosis. The long nonabsorbable monofilament sutures helped in localization of the rectal stump [Figure 1]. A 5 mm port was sometimes required in the suprapubic area to facilitate the adhesiolysis, mobilization and anastomosis. The splenic flexure was mobilized in all the patients. The colostomy was fully mobilized extracorporeally and was helped intracorporeally by adhesiolysis around it. The anvil was inserted in the proximal colon through the colostomy and the proximal colon was returned back to the abdominal cavity [Figure 2]. The colostomy wound was closed and this wound was utilized for the 10-mm port [Figure 3 and 4]. An end-to-end intracorporeal anastomosis was performed by circular stapler (proximate ILS size 29, Johnson and Johnson). The shaft of the circular stapler was inserted through the rectal stump and engaged into the anvil in the proximal colon [Figure 5]. Finally the pelvic cavity was filled with saline and the air leak test was done by insufflating the air in the rectum while visualizing the anastomosis by the sigmoidoscope at the same time. A through lavage was done on completion of the procedure.

## RESULTS

There was no anastomotic dehiscence, postoperative complications and mortality. Two patients in whom the procedure could not be completed laparoscopically were converted to open. No patient required blood transfusion during the surgery. Patients were allowed clear fluids from the second postoperative day. The normal bowel activity was achieved within 3 to 5 days. No patient required a temporary colostomy or an ileostomy. There was less postoperative pain, probably because of minimal wounding. No complications were reported in 6 months to 3 years of follow-up. The hospital stay was from 4-11 days (mean 7 days).

## DISCUSSION

The Hartmann procedure is usually done as a temporary emergency procedure for the left-sided colonic pathology when conditions are not ideal for a primary anastomosis. It is a fast and safer operation in adverse general status and local abdominal conditions. A large number of these patients have associated medical comorbidities. Although done mostly for the perforated diverticular disease, it can serve as a permanent colostomy, especially in patients with obstructing rectal carcinoma of middle and upper third. It has also been accepted as a curative procedure in emergency for obstructive and perforated left colonic malignancy. It has the advantage of removing the diseased bowel at the first stage with no risk of primary anastomotic leak, thereby eliminating anastomotic, perineal and functional problems. Role of Hartmann procedure as an emergency surgery is controversial as several therapeutic alternatives have emerged, relegating its use. On table, lavage with primary anastomosis, primary anastomosis with proximal diversion ileostomy and primary anastomosis with proximal diversion colostomy have given better results in terms of morbidity and mortality compared to Hartmann procedure.[3–5] The morbidity and mortality of Hartmann procedure is higher than these therapeutic alternatives due to the fact that it is done on poor-risk patients and by the junior-duty surgeons in the face of diffuse peritonitis. The primary anastomosis and Hartmann procedure are not competing operations but are situation-dependent. There are no prospective randomized studies comparing both therapeutic options and these two therapeutic options fit different groups of patients whose disease differ with respect to etiology, localization and severity. Armbruster *et al* have analyzed the literature of the past few years and have defined some criteria to decide when to exclude primary anastomosis: MPI>20, APACHE II score >15, preoperative organ insufficiency, Hinchey grade III or IV and ASA score IV.[6] Despite the documented morbidity and mortality associated with its reversal, HP remains a favored procedure in emergency situations where primary anastomosis is considered unsafe.

The subsequent restoration of intestinal continuity is desirable to improve the quality of life but can be technically challenging. The optimal timing for the reversal is controversial, but the operative difficulties appear to be less after a delay of 15 weeks. Conventionally, the reversal was done by the open method typically requiring a laparotomy and was associated with considerable morbidity and mortality and one-third of the patients were left with permanent colostomy. There is also reluctance on the part of surgeons for this potentially difficult but avoidable operation. The advance in laparoscopy and stapler technology is changing the attitude of surgeons and has made this reversal safer and easier, with an increase in the reversal percentage.

### Technical issues

There are several laparoscopic techniques of reversal of Hartmann procedure. The principle common to all techniques is a tension-free intracorporeal stapler anastomosis. This is ensured by the mobilization of the splenic flexure and the division of left colonic vessels, as we have done in all of our patients. In case of colonic resection, the specimen can be brought out by a small left lower quadrant incision or a transverse suprapubic incision. We have done a diagnostic laparoscopy, first, by the introduction of lateral ports to assess the severity of adhesion and to assess the rectal stump. This has helped us in making decision regarding the feasibility of laparoscopic reversal. The introduction of circular stapler in the rectal stump helps in identification and mobilization of the rectal stump. Others have mobilized the colostomy first and have used the colostomy site as a first port or used a standard umbilical port.[7] Lucarini *et al* have done the reversal by laparoscopic-assisted method using the Dexterity Pneumo Sleeve device and minilaparotomy for the colostomy mobilization.[8] Jacob *et al* have demonstrated in a canine model that it has been possible to reverse the colostomy, 1 week postoperatively, using a dual endoscopic-assisted computer-mediated circular stapling device.[9] Others have used circular anastomotic device in which the bowel lumen remain closed to minimize the contamination. Bossotti *et al* have described a novel technique of gasless laparoscopic-assisted colostomy closure, which is safer on elderly patients with cardiovascular diseases; reduces trauma, postoperative pain, bacterial contamination; and with this technique, it is possible to use the traditional instruments with reduction in cost of operation.[10]

### Result

Laparoscopic reversal has shown lesser morbidity and mortality. The published results and literature review have shown that compared to open reversal, there was less intraoperative blood loss, shorter hospital stay, less wound infection rate, less postoperative pain and lower incidence of pelvic abscess, anastomotic leak and incisional hernia. The patient convalescence, the first evacuation and oral feeding are achieved faster.

### Merits

Laparoscopically the reversal rate is higher and the procedure is safer and easier. The introduction of circular stapler and advance in technology have made the reversal possible for older patients in high-risk group. The incidence of anastomotic leak is lower than in open procedure. The adhesiolysis and localization of rectal stump are easier laparoscopically than by open method.

### Limitations

It is technically challenging and requires an experienced laparoscopic surgeon but offers clear advantages to patients. Main reasons reported for conversion to open were dense abdominal-pelvic adhesions secondary to diffuse peritonitis at the primary operation, short delay before the reconstruction, difficulty in finding the rectal stump and rectal scarring. Leaving long nonabsorbable suture ends at the rectal stump or suturing it to the anterior abdominal wall helps in its localization. Other relative limitation factors could be a large incisional hernia from the previous laparotomy and contraindications to general anesthesia and laparoscopy.

## CONCLUSION

The reversal of Hartmann procedure can be difficult due tendency of Hartmann segment to become densely adherent deep in the pelvis. The laparoscopic reversal has made this major operation easier, safe and practical. As a majority of these patients is in the elderly age group, it has the advantage of early mobilization, less pain, short hospital stay and return to normal life.
